# Characterization of Blue Light Receptors LreA and LreB in *Aspergillus flavus*

**DOI:** 10.4014/jmb.2411.11054

**Published:** 2025-02-14

**Authors:** Hye-Min Park, Ye-Eun Son, He-Jin Cho, Jae-Hyuk Yu, Hee-Soo Park

**Affiliations:** 1 School of Food Science and Biotechnology, Kyungpook National University, Daegu 41566, Republic of Korea; 2 Department of Bacteriology, University of Wisconsin-Madison, Madison, WI 53706, USA; 3 Department of Integrative Biology, Kyungpook National University, Daegu 41566, Republic of Korea

**Keywords:** *Aspergillus flavus*, light receptors, LreA, LreB, asexual development

## Abstract

Light is a key external signal factor that regulates asexual development, stress resistance, and secondary metabolism in fungi. In the presence of light, photoreceptors sense several light receptors and affect fungal life. In this study, we characterized the function of the blue light receptors LreA and LreB in *Aspergillus flavus*, a potent pathogenic and toxigenic fungus. *lreA* or *lreB* deletion increased the growth rate but decreased conidial production in the presence or absence of light. The Δ*lreA*-mutant strain and the Δ*lreB*-mutant strain produced abnormal conidiophores, suggesting that *lreA* and *lreB* were essential for proper conidiation in *A. flavus*. The absence of *lreA* or *lreB* slightly decreased the stress response tolerance against thermal and oxidative stresses. In kernel infection, the Δ*lreA* mutant strain and the Δ*lreB* mutant strain produced conidia and aflatoxin B1 that were less than those produced by the control strains. Therefore, LreA and LreB play key roles in the growth, asexual development, and pathogenicity of *A. flavus*.

## Introduction

*Aspergillus flavus* is a representative mycotoxin-producing and pathogenic filamentous fungus that substantially affects humans [1-3]. It produces mycotoxins called aflatoxins, which can cause cancer in humans if they contaminate food [4, 5]. It infects plants or stored grains, causing economic losses [1, 6]. When its asexual spores infect immunocompromised people, this species can cause aspergillosis, which is life threatening [1]. *A. flavus* reproduces through asexual or sexual development, but its reproduction mainly occurs by producing asexual spores called conidia. Asexual reproduction is complex, but it is precisely regulated by various external environmental factors and internal regulators [2, 7, 8].

Fungal development is affected by various environmental cues, such as light, gas, and humidity [9, 10]. Among them, light is an important external environmental factor affecting fungi and other species; for example, it affects photosynthesis in plants [11, 12]. Fungi have photoreceptors that recognize blue, green, and red light wavelengths [10, 13]. Studies have explored blue light receptors, which have four types: white collars, vivid, blue F protein, and cryptochrome/photolyase [14]. Green light receptors include opsin, but it is not clearly identified; the red light receptor is a fungal phytochrome [14, 15]. Each light sensor contains chromophores [16], which are light-absorbing chemical groups, including retinal, flavin, and biliverdin for blue, green, and red light sensors, respectively [14, 15].

Previous studies characterized various fungal eyes in fungi [14, 17]. In *Neurospora crassa*, blue light receptors are named white collars (WC), namely, WC1 and WC2, because each deletion mutant do not show photoinduced carotenogenesis [18-20]. WC1 and WC2 function by forming a white collar complex [21]. They act as transcription factors that regulate the gene expression related to circadian rhythm and carotenoid biosynthesis [22, 23]. Light response A (LreA) and light response B (LreB), which are homologs of WC1 and WC2, respectively, have been studied for the repressed mycotoxin formation in *A. nidulans* [24]. FphA participates in sexual development and mycotoxin formation in *A. nidulans* [24, 25] and regulates germination in *A. fumigatus* [26]. However, the roles of light receptors in *A. flavus* are less described.

*A. flavus* has several photoreceptors that perceive different light wavelengths. It contains LreA and LreB, which sense blue light; FphA, which senses red light; opsins, which detect green light; and cryptochrome, which recognizes ultraviolet light [10]. In the present study, we characterized LreA and LreB in *A. flavus*. To investigate their role, we generated the deletion mutants of *lreA* (*AFLA_010605*) and *lreB* (*AFLA_011062*). We then performed a phenotypic analysis.

## Materials and Methods

### Strains, Media, and Cultural Conditions

The fungal strains used in this study are listed in Table 1. Under general experimental conditions, each *A. flavus* strain was cultured on solid minimal media with 1% glucose (MMG: 5% nitrate salt solution composed of 120 g/l NaNO_3_, 10.4 g/l KCl, 10.4 g/l MgSO_4_·H_2_O, 30.4 g/l KH_2_PO_4_, and 0.1% trace element solution [pH 5.5] composed of 22 g/l ZnSO_4_∙7H_2_O, 11 g/l H_3_BO_3_, 5 g/l MnCl_2_∙4H_2_O, 5 g/l FeSO_4_∙7H_2_O, 1.6 g/l CoCl_2_∙5H_2_O, 1.6 g/l CuSO_4_·5H_2_O, 1.1 g/l (NH_4_) _6_Mo_7_O_24_∙4H_2_O, 50 g/l Na_2_EDTA, and 10 g/l glucose; pH 6.5) with 0.1% yeast extract (MMGY) [27]. The strains were incubated at 37°C. Depending on the characteristics of the strain, uridine and uracil were added. They were grown under light conditions to observe asexual development and under dark conditions to observe sexual development. *A. flavus* strains were cultured on a liquid complete medium (CM; 20 g/l glucose, 5% nitrate salt solution, 0.1% trace element solution pH 5.5, 1.5 g/l casamino acids, 2 g/l bacto-peptone; pH 6.5) at 30°C for 7 days to confirm their toxin production ability [28]. For several osmotic stimulations, 0.75 M KCl, 0.75 M NaCl, and 0.75 M sorbitol were added to the MMGY medium and cultured under light conditions for 5 days.

### Construction of *lreA* and *lreB* Deletion Mutant Strains

The oligonucleotides used in this study are listed in Table 2. Double-joint PCR (DJ-PCR) was utilized to produce deletion mutant strains [29]. DNA cassettes, including 5' (DF-TailR) and 3' (TailF-DR) sides of the target gene, were amplified using the genomic DNA (gDNA) of *A. flavus* NRRL3357 as a template. The *pyrG* region of *A. fumigatus* was amplified using 5'_*AfupyrG*_F/3'_*AfupyrG*_R as primers. Then, three PCR products were connected and amplified using the NF/NR primers to obtain the final DNA fragment. The *A. flavus* NRRL3357.5 strain was used as the host strain to construct the deletion mutants of *lreA* and *lreB*. The final DNA fragment was added to the protoplasts of *A. flavus* NRRL3357.5 made with Vinoflow FCE lysing enzyme (Novozymes, Denmark) [30]. Transformants were grown on a solid MMGY medium without uracil and uridine. The gDNA of each transformant was extracted and used as a PCR template to verify the deletion mutant strains.

### Generation of *lreA*- and *lreB*-Complemented Strains

The PCR cassettes with the predicted promoter sequence and open reading frame (ORF) region of each gene were amplified using primers to generate complemented strains (Table 2). pYES1 [31] and the PCR cassettes were treated with the *Not*Ι restriction enzyme and then combined [32]. The combined plasmids, namely, pHM 1.1 (with the *lreA* promoter sequence and ORF region) and pHM 2.1 (with the *lreB* promoter region and ORF sequence), were introduced to the protoplasts of each deletion mutant. PCR confirmation and restriction enzyme treatment were performed to confirm whether it was a complemented strain.

### Analysis of Conidial Production Ability

Each strain was inoculated on solid MMGY media and grown at 37°C for 5 days under dark and light conditions to confirm conidial production ability. Conidia were collected using ddH_2_O with 0.01% Triton X-100 (Sigma, USA) and filtered through miracloth (Calbiochem, USA) to collect pure conidia. The number of conidia was counted using a hematocytometer. The number of conidia of each strain was counted in triplicate.

### Quantitative Reverse Transcription PCR (qRT-PCR)

Silica beads and RIboEX were added to the prepared samples, fresh conidia, or squeezed mycelia to isolate total RNA [33, 34]. They were homogenized with a mini bead beater. After chloroform was added, the homogenized sample was centrifuged to separate the aqua phase. Equal amounts of supernatant and isopropanol were added, and the RNA pellet was washed with ethanol (70%) with diethylpyrocarbonate (DEPC)-treated water. The RNA pellet was dissolved in RNase-free water and treated with DNase I (Promega, USA) for 30 min. DNase was inactivated with DNase stop buffer (Promega). After the purity of RNA was evaluated, it was used for cDNA synthesis or stored at −80°C.

cDNA was synthesized at a constant concentration by using total RNA. The same concentration of cDNA was synthesized with total RNA by using GoStript reverse transcriptase (Promega). Real-time PCR was performed using iTaq Universal SYBR Green Supermix (Bio-Rad, USA) with CFX96 Touch Real-Time PCR (Bio-Rad). The *β*-actin gene was used as an endogenous control.

### Stress Tolerance Assay

A stress resistance test was performed using previously described methods [34, 35] with some modifications. Three-day-old fresh conidia of each strain were collected and diluted to prepare 10^3^ spores/ml for heat stress examination. The diluted conidial suspension was incubated at 55°C for 15 min, and untreated conidia were used as controls. The viability of conidia was measured by counting the number of colonies formed after the conidial suspension was inoculated and cultured. Then, 0.05 M H_2_O_2_ was added to 10^3^ conidia of each strain and incubated at 37°C for 30 min to investigate the oxidative stress resistance of conidia. Stress tolerance was determined by counting the conidia grown by incubating 100 spores from the treated and untreated conidia. Stress tolerance tests were performed in triplicate.

### Kernel Assay and Aflatoxin B1 Production Assay

A kernel assay was performed to confirm the pathogenicity of each strain in accordance with previously described methods [34, 36]. *Zea mays* L. VSC03 (Asia Seed Co., Ltd., Republic of Korea) kernels were soaked in 70% ethanol and shaken strongly using a RotoBot mini programmable rotator (Benchmark Scientific, USA). After being drenched in 2% sodium hypochlorite, the soaked kernels were shaken with a rotator and rinsed with ddH_2_O. The washed kernels were wound with sterile needles (Dawonscience, Republic of Korea). Each kernel was infected with the conidia (5 × 10^5^ spores) of each strain. The infected kernels were cultured at 30°C for 7 days under light conditions. The number of conidia was counted using a hemocytometer to examine conidiation. Chloroform was added to each sample and incubated overnight to extract aflatoxin B1 from kernels. After incubation, the chloroform phase was collected and evaporated. In the dried sample, 100 μl of chloroform was added and loaded into the TLC plate. The TLC plate was placed in chloroform: ethyl acetate (Daejung, Republic of Korea) (9:1, v/v) and photographed under UV exposure (366 nm). Band intensity was measured using ImageJ software.

### Microscopy

Photos were taken with a Pentax MX-1 digital camera. Micrographs were filmed using a Zeiss Lab.A1 microscope equipped with an AxioCam 105c camera and AxioVision (Rel. 4.9) digital imaging software.

### Statistical Analysis

Data were statistically analyzed using GraphPad Prism Version 5.01 software. The statistical differences shown in the graph were determined through ANOVA with Tukey’s test. Data were presented as mean ± standard deviation (SD). Statistically significant differences were set at *p* < 0.05 (*p ≤ 0.05; **p ≤ 0.01; ***p ≤ 0.001)

## Results

### LreA and LreB in *A. flavus*

**Fig. 1:**
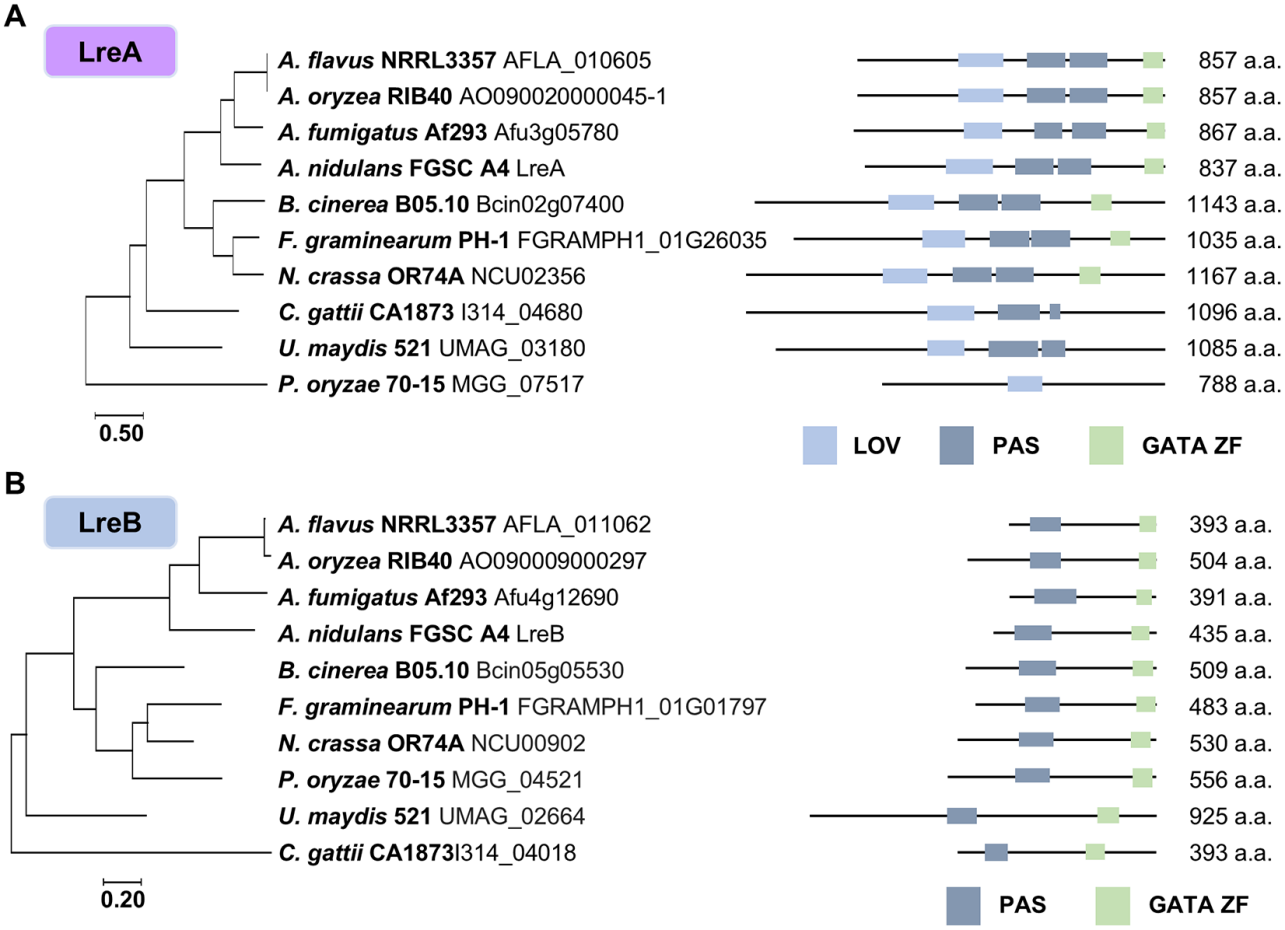
Domain analysis of LreA and LreB in *Aspergillus flavus*. (**A–B**) Phylogenetic trees and domain analysis of LreA and LreB in several fungi. Each light receptor was conserved in several fungal species. The phylogenetic trees show the following fungal species: *A. flavus* NRRL3357; *A. oryzea* RIB40; *A. fumigatus* Af293; *A. nidulans* FGSC A4; *B. cinerea* B05.10, *Botrytis cinerea* B05.10; *F. graminearum* PH-1, *Fusarium graminearum* PH-1; *N. crassa* OR74A, *Neurospora crassa* OR74A; *C. gattii* CA1873, *Cryptococcus gattii* CA1873; *U. maydis* 521, *Ustilago maydis* 521; *P. oryzae* 70-15, *Pyricularia oryzae* 70-15 (synonym *Magnaporthe oryzae* 70-15). The following domains are shown in the phylogenetic trees: LOV, light, oxygen, and voltage; PAS, per, arnt, sim; GATA ZF, GATA zinc finger.

**Fig. 2:**
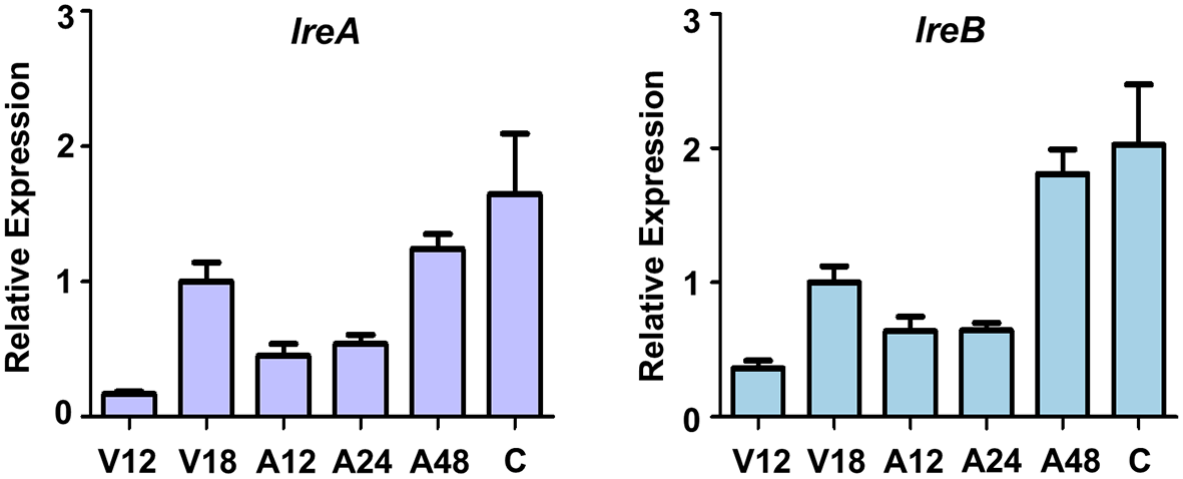
Levels of *lreA* and *lreB* in *A. flavus* life cycle. The life cycles of *lreA* and *lreB* and in *A. flavus*. Samples from each growth stage were collected, mRNA was extracted, and qRT-PCR was performed using primers for each of the three genes. (V; vegetative development, A; asexual growth).

The protein sequences of LreA and LreB of 10 fungal species were obtained from Fungi DB (https://fungidb.org) to confirm that light receptors are conserved in various fungal species. Based on these sequences, phylogenetic trees were generated using MEGA11 software (https://www.megasoftware.net/). Domain analysis was performed for each species by using InterPro software (https://www.ebi.ac.uk/interpro/). LreA and LreB are conserved in various fungal species (Fig. 1). LreA contains a light-oxygen-voltage-sensing domain (LOV domain), Per-Arnt-Sim (PAS) domains, and a GATA zinc finger (ZF) domain (Fig. 1A). PAS domains play a role in protein–protein interactions [37]. LreB has a PAS domain and a GATA ZF domain in all the analyzed fungi (Fig. 1B). The transcript levels of *lreA* and *lreB* during the life cycle were measured via qRT-PCR (Fig. 2). In the life cycle of *lreA* and *lreB*, their mRNA expression levels, excluding conidia, were high 48 h after the induction of asexual development. The mRNA expression levels of *lreA* and *lreB* were also high in the conidia throughout their life cycle.

### Roles of LreA and LreB in Fungal Growth and Development

**Fig. 3:**
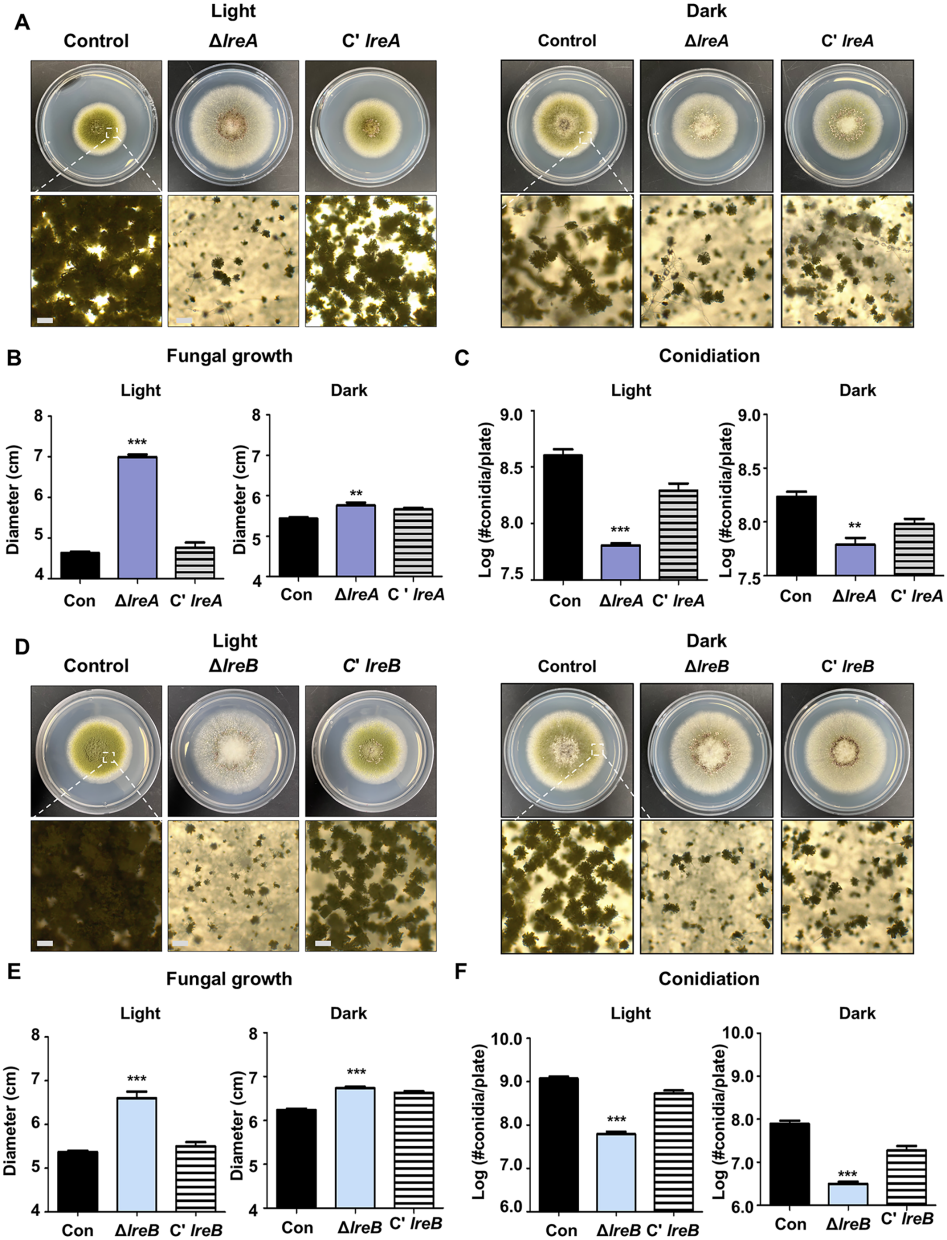
Phenotypic analysis of *lreA*-deletion mutant and *lreB*-deletion mutant in *A. flavus*. (**A**) Phenotypes of the Δ*lreA* strain grown at 37°C under light and dark conditions for 5 days. (**B**) Colony diameter of the Δ*lreA* strain under light and dark conditions. (Control vs. Δ*lreA*, **p ≤ 0.01; ***p ≤ 0.001, *n* = 3). (**C**) Conidial production ability of the Δ*lreA* strain under light and dark conditions. (Control vs. Δ*lreA*, **p ≤ 0.01; ***p ≤ 0.001, *n* = 3). (**D**) Phenotypes of Δ*lreB* grown at 37°C under light and dark conditions for 5 days. (**E**) Colony diameter of the Δ*lreB* strain under light and dark conditions (control vs. Δ*lreB*, ***p ≤ 0.001, *n* = 3). (**F**) Conidial production ability of the Δ*lreB* strain under light and dark conditions (control vs. Δ*lreB*, ***p ≤ 0.001, *n* = 3).

The deletion mutant and complemented strains of *lreA* (*AFLA_010605*) and *lreB* (*AFLA_011062*) were generated to investigate the roles of LreA and LreB. Each strain was point-inoculated on a solid MMGY medium and cultured at 37°C for 5 days under both light and dark conditions (Fig. 3). The growth of the Δ*lreA* strains increased compared with that of the control (Fig. 3A and 3B). The number of conidia in the Δ*lreA* strains was reduced compared with that in the control (Fig. 3C). The growth of the Δ*lreB* strains increased, but the conidial production of this mutant decreased under both light and dark conditions (Fig. 3D–3F).

**Fig. 4:**
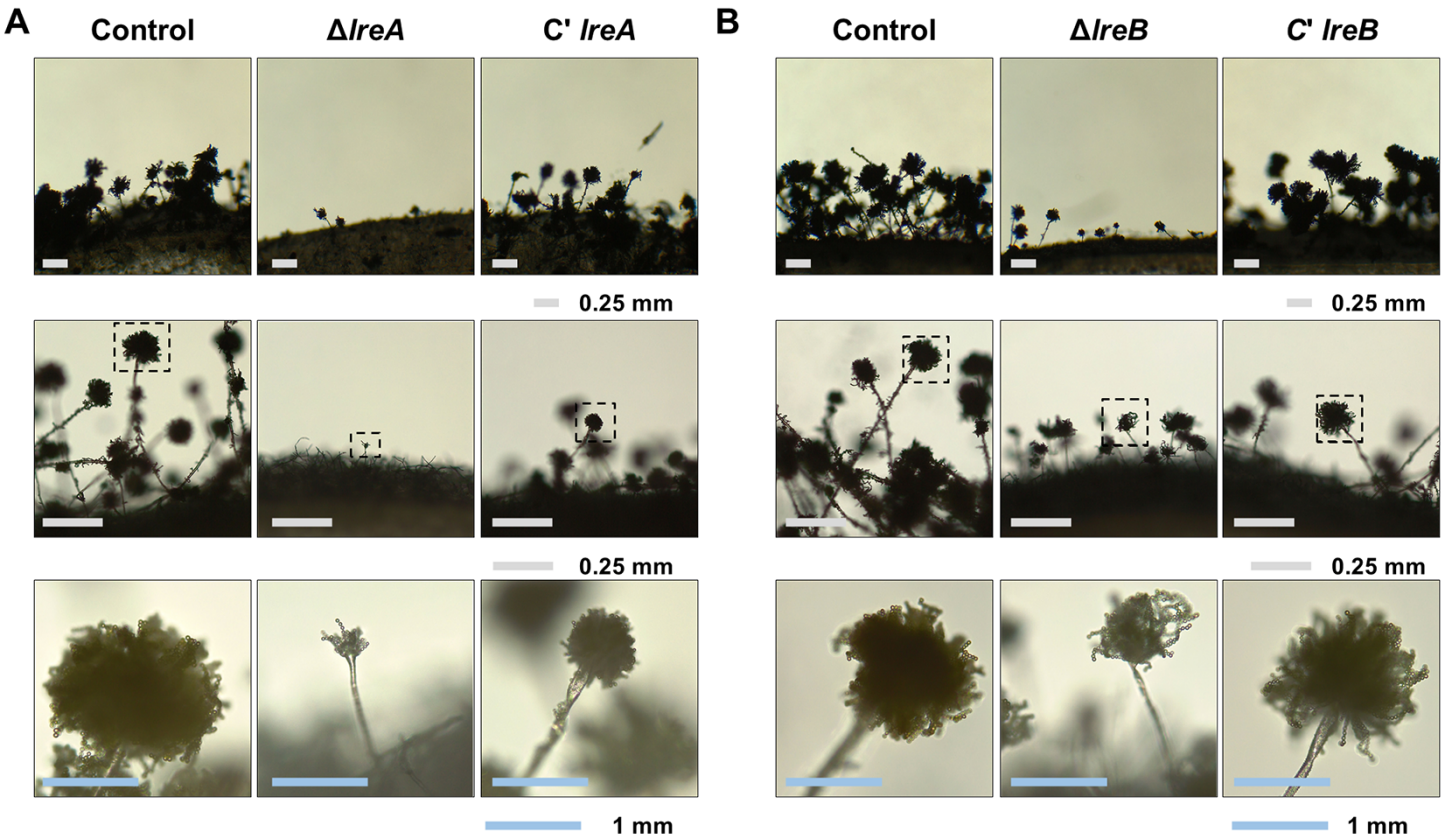
Effects on the conidiophore formation of LreA and LreB in *A. flavus*. (**A**) Morphological characteristics of conidiophores in the control, Δ*lreA*, and C'*lreA* strains cultured by inducing asexual development under light conditions for 2 days. Middle and bottom panels are close-up photographs of the conidiophores of each strain. (**B**) Conidiophore appearances in the control, Δ*lreB*, and C'*lreB* strains cultured by inducing asexual development under light conditions for 2 days. Middle and bottom panels are close-up photographs of the conidiophores of each strain.

Each strain was inoculated in an agar block and cultured to induce asexual development by exposing them to light at 37°C for 2 days and further examine the roles of LreA and LreB in conidiation (Fig. 4). The conidiophores of the Δ*lreA* mutant strain and the Δ*lreB* mutant strain were abnormal and lacking conidia (Fig. 4). Therefore, LreA and LreB likely played crucial roles in fungal growth and asexual development.

Asexual development is induced not only by light but also by osmotic stimulation and aeration [38, 39]. The Δ*lreA* mutant strain and the Δ*lreB* mutant strain were exposed to osmotic stress compounds to determine whether the decrease in conidial production was caused by the deletion of *lreA* or whether *lreB* gene deletion was rescued by other inducers (Fig. S1). The conidial production of the Δ*lreA* mutant strain and the Δ*lreB* mutant strain in the MMGY medium containing 0.75 M KCl, 0.75 M NaCl, or 0.75 M sorbitol increased compared with that in the MMGY medium. These results suggested that light and osmotic stimulation had an independent asexual development-inducing pathway.

To examine the roles of LreA and LreB in sexual development, we also examined sclerotium production. Fig. S2 shows that the absence of *lreA* or *lreB* increased sclerotium production, suggesting that LreA and LreB are required for proper sexual development in *A. flavus*.

### Function of LreA and LreB in Conidial Stress Tolerance

**Fig. 5:**
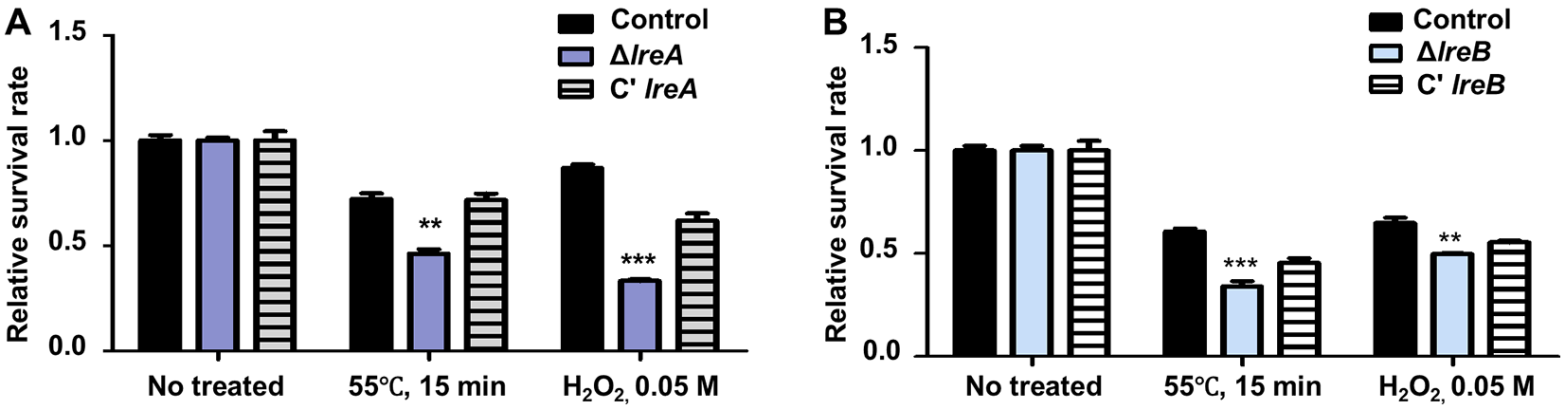
Stress tolerance assay of *lreA*- and *lreB*-null mutant conidia in *A. flavus*. (**A**) Relative survival rates of the Δ*lreA* conidia exposed to heat stress (55°C for 15 min) or oxidative stress (0.05 M H_2_O_2_). (**B**) Survival rates of the Δ*lreB* conidia exposed to heat stress (55°C for 15 min) or oxidative stress (0.05 M H_2_O_2_; control vs. mutants, *p ≤ 0.05; **p ≤ 0.01; ***p ≤ 0.001, *n* = 3).

The mRNA levels of *lreA* and *lreB* in the conidia were high (Fig. 2), suggesting that LreA or LreB was essential for conidia. To verify this suggestion, we examined the conidial viability and germination, but we found no difference between the control and the Δ*lreA* mutant conidia and the Δ*lreB* mutant conidia. We then analyzed the stress tolerance of the Δ*lreA* mutant conidia and the Δ*lreB* mutant conidia. The Δ*lreA* mutant conidia or the Δ*lreB* mutant conidia were more sensitive against thermal and oxidative stresses than the control (Fig. 5). Therefore, LreA and LreB were involved in stress tolerance.

### Pathogenicity of the *lreA* Single Deletion Mutant and the *lreB* Single Deletion Mutants

**Fig. 6:**
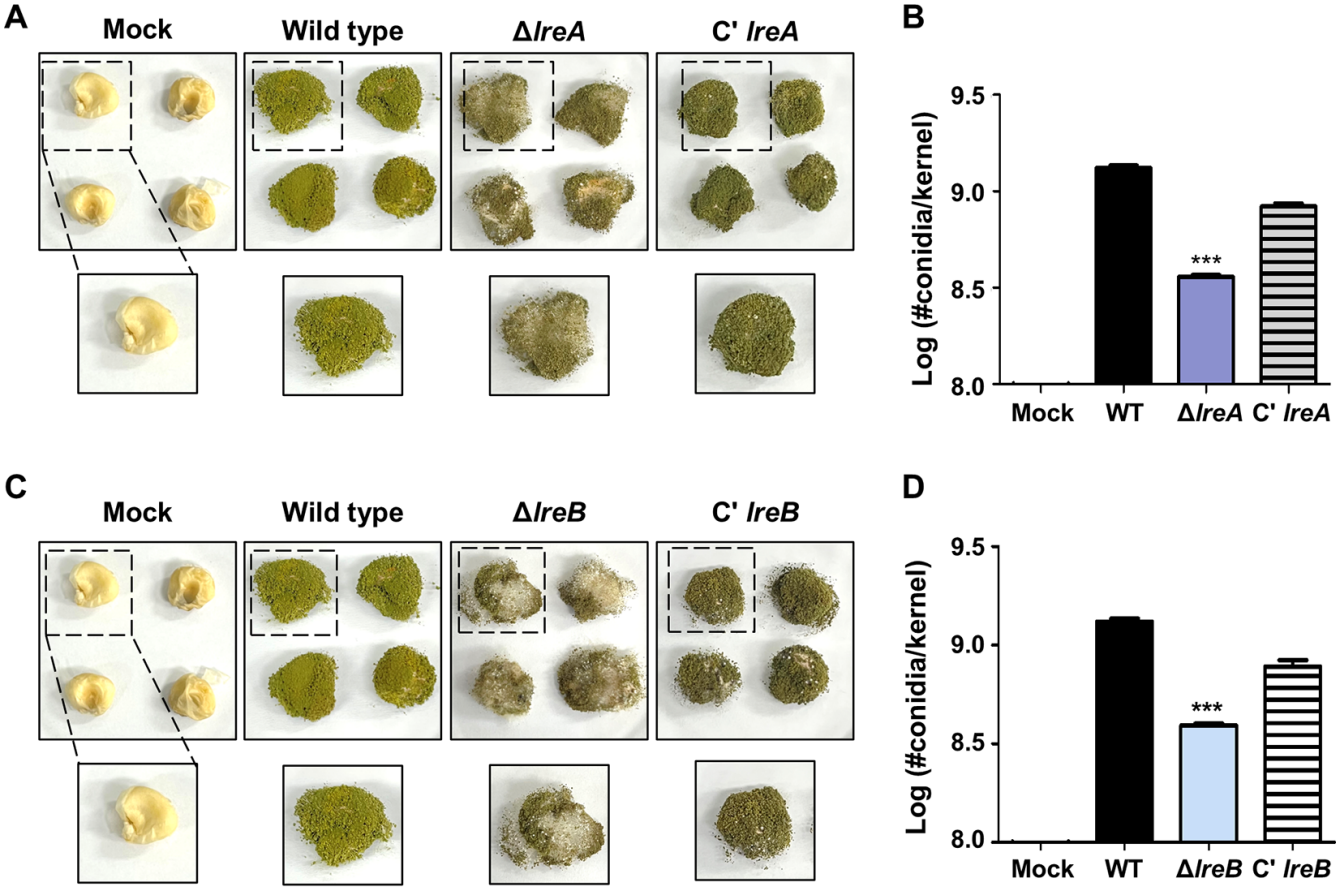
Role of *lreA* and *lreB* in kernel infection. (**A**) Results obtained after growth at 30°C under light conditions for 7 days following kernel infection with the wild-type, Δ*lreA*, and C'*lreA* conidia. The mock refers to the kernel itself, which was not inoculated with any conidial strain. The others were inoculated with the wild-type, Δ*lreA*, and C'*lreA* strains. (**B**) Counting and comparison of the number of conidia growing on each maize kernel for 7 days (control vs. Δ*lreA*, ***p ≤ 0.001, *n* = 3). (**C**) These results were obtained after the kernels were infected with the wild-type, Δ*lreB*, and C'*lreB* conidia and grown at 30°C under light conditions for 7 days. The mock represents uninfected kernels, while the others were grown with the inoculation of the wild-type, Δ*lreB*, and C'*lreB* conidia for 7 days. (**D**) The conidia of each strain were cultured on kernels for 7 days, and fungal colonization was compared (Control vs. Δ*lreB*, ***p ≤ 0.001, *n* = 3).

Kernel assays were performed using maize kernels to determine whether LreA and LreB contributed to fungal pathogenicity. The kernels inoculated with the conidia of each strain were inoculated in maize kernels and cultured at 30°C under light conditions for 7 days (Fig. 6). The number of conidia grown on kernels decreased in the Δ*lreA* strains compared with that in the control (Fig. 6A and 6B). The same experiment was performed by inoculating the kernels with equal amounts of conidia from each strain for 14 days. The Δ*lreA* strains exhibited a decreased ability to produce conidia on kernels compared with the control (Fig. S3A and S3B). The number of conidia in the Δ*lreB* strains decreased compared with that in the control strain after 7 (Fig. 6C and 6D) and 14 days of incubation (Fig. S3C and S3D). Overall, these results indicated that LreA and LreB positively regulated the fungal pathogenicity of maize.

### Aflatoxin B1 Production of *lreA* and *lreB* Deletion Mutants

**Fig. 7:**
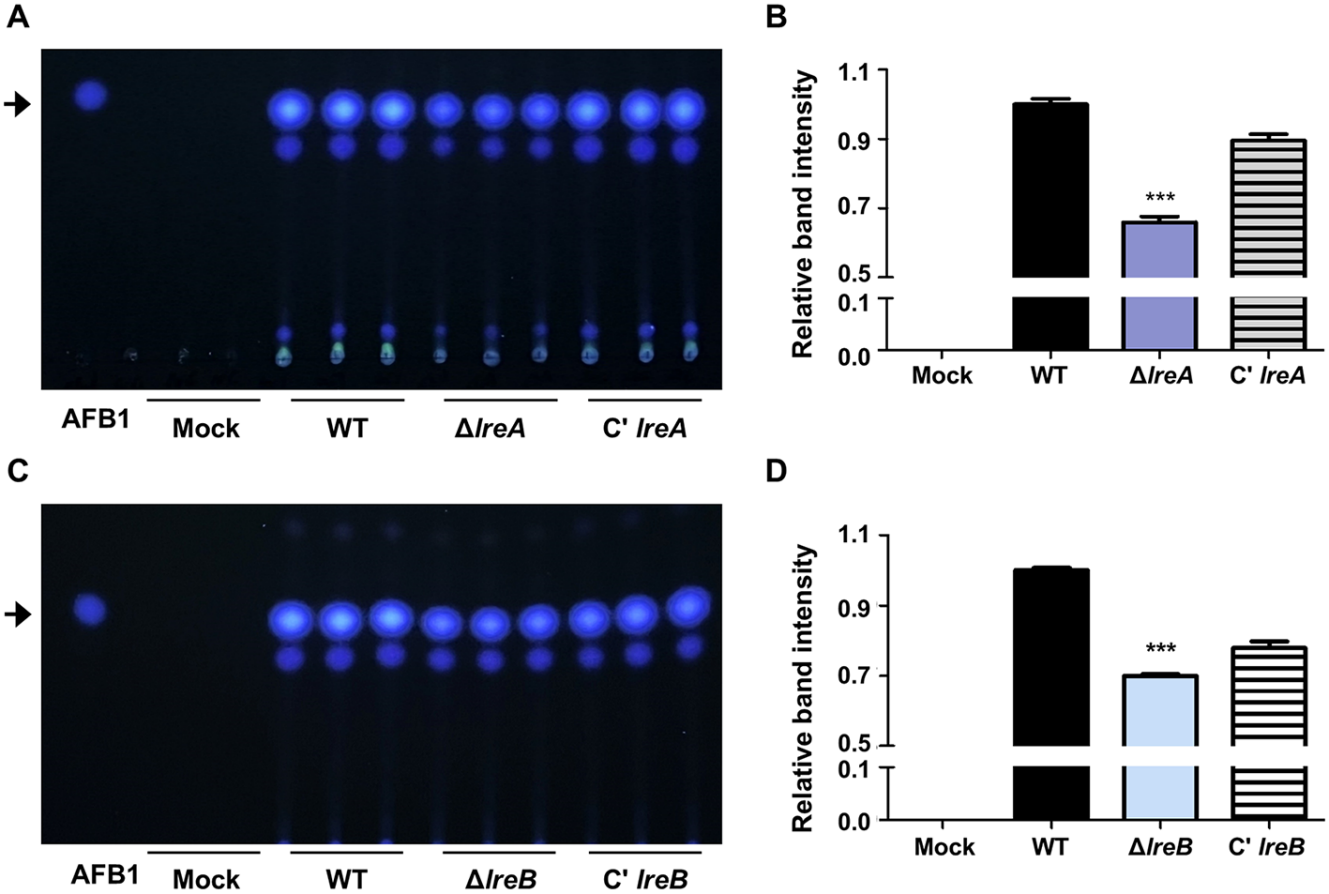
Influence of *lreA* and *lreB* in aflatoxin B_1_ production in kernel infection. (**A**) Image showing the production of aflatoxin B_1_ extracted from the kernel samples infected with control, Δ*lreA*, and C'*lreA* conidia for 7 days of cultivation. (**B**) Relative band intensity of aflatoxin B_1_ according to the TLC result in (**A**). Error bars indicate the standard error of the mean from three biological replicates (WT vs. Δ*lreA*, ***p ≤ 0.001, *n* = 3). (**C**) Image showing the production of aflatoxin B_1_ extracted from the kernel samples infected with control, Δ*lreB*, and C'*lreB* conidia for 7 days of cultivation. (**D**) Relative band intensity of aflatoxin B_1_ according to the TLC result in (**C**). Error bars indicate the standard error of the mean from three biological replicates (WT vs. Δ*lreB*, ***p ≤ 0.001, *n* = 3).

We examined the aflatoxin B1 production of the Δ*lreA* mutant strain and the Δ*lreB* mutant strain in maize. We found that the amount of aflatoxin B1 produced by the Δ*lreA* mutant strain and the Δ*lreB* mutant strain was less than that produced by the control or complemented strains after 7 days of incubation (Fig. 7). After 14 days of incubation, aflatoxin B1 was confirmed in the kernel infected with the Δ*lreA* mutant strain or the Δ*lreB* mutant strain, and its amount was lower than that in the kernel infected with the control or complemented strains (Fig. S4).

## Discussion

Among various external factors, light remarkably influences fungal life, including developmental patterns [40]. In *A. nidulans*, LreA and LreB affect sexual development but not asexual development [24]. In *A. fumigatus*, the absence of *lreA* decreases conidiation under light conditions [41, 42]. In our study, the Δ*lreA* mutant strain and the Δ*lreB* mutant strain produced less amount of conidiation in *A. flavus* (Fig. 3). These results suggested that LreA and LreB participated in asexual formation in *A. fumigatus* and *A. flavus* but not in *A. nidulans*. Similar to asexual development, sexual reproduction involves LreA and LreB with different roles between *A. nidulans* and *A. flavus*. In *A. nidulans*, the deletion of *lreA* and *lreB* decreased cleistothecium formation under light and dark conditions. In *A. flavus*, the Δ*lreA* mutant strain and the Δ*lreB* mutant strain produced more sclerotia than the control strain did (Fig. S2). These results implied that LreA and LreB play diverse roles in the development of *A. flavus* and *A. nidulans*.

Light affects fungal development and secondary metabolism [40, 43]. In some fungi, light activates the production of specific secondary metabolites [43]. However, in other cases, light represses the production of secondary metabolites; for example, sterigmatocystin production in *A. nidulans* increases under dark conditions [44, 45]. This process is light-dependently controlled by various regulators, including VeA, VelB, LaeA, and LreA [43]. In *A. nidulans*, the deletion of *lreA* or *lreB* decreases the production of sterigmatocystin, which is closely related to aflatoxins [24]. Our study found that the absence of *lreA* or *lreB* decreased aflatoxin B1 production in *A. flavus* (Fig. 6). Therefore, LreA and LreB are key roles in secondary metabolism in *Aspergillus* species.

In conidia, the mRNA expression levels of *lreA* and *lreB* are high in the life cycle (Fig. 2), indicating that LreA and LreB may play a key role in conidia. We checked the germination rate of the Δ*lreA* mutant conidia and the Δ*lreB* mutant conidia, but the control and mutant strains did not differ (data not shown). This result is similar to that observed in *A. fumigatus* [41], suggesting that LreA and LreB do not affect fungal germination. We also examined the conidial viability, the amount of conidial trehalose, and the degree of conidial stress tolerance. We found that the deletion of *lreA* and *lreB* did not influence conidial viability (data not shown); instead, it altered the degree of conidial trehalose and conidial stress tolerance (Fig. 5). These results demonstrated that LreA and LreB participate in conidial stress response, but the detailed molecular mechanism of these regulators remains unknown. Further studies should be performed to verify the roles of LreA and LreB in stress response.

In conclusion, LreA and LreB proteins are crucial for conidiophore formation, sclerotium production, and conidial stress tolerance. The deletion of *lreA* and *lreB* affects plant pathogenesis and aflatoxin B1 production. These results suggest the physiological roles of LreA and LreB in *A. flavus*. However, the detailed molecular mechanisms and direct targets of LreA and LreB remain unknown and warrant further investigations.

## Supplemental Materials

Supplementary data for this paper are available on-line only at http://jmb.or.kr.



## Figures and Tables

**Table 1:** *Aspergillus flavus* strains used in this study.

Strains	Relevant genotype	References
NRRL3357	Wild-type *A. flavus*	ATCC
NRRL3357.5	*A. flavus* *pyrG*^-^	[46]
TTJ6.1	*A. flavus* *pyrG*^-^; *AfupyrG*^+^	[47]
THM1.1~3	*pyrG*^-^; Δ*lreA::AfupyrG*^+^	This study
THM2.1~3	*pyrG*^-^; Δ*lreB::AfupyrG*^+^	This study
THM3.1	*pyrG*^-^; Δ*lreB*::*AfupyrG*^+^; *lreB* (*p*)::*lreB::FLAG4x::ptrA*	This study
THM11.1	*pyrG*^-^; Δ*lreA::AfupyrG*^+^; *lreA* (*p*)*::lreA::FLAG4x::ptrA*	This study

**Table 2:** Oligonucleotides used in this study.

Name	Sequence (5' → 3')	Description
OHS1542	CCTGGTCTTTGGTTTGGTACACC	*AfupyrG* _F
OHS1543	CGACTGGCAGGAGATGATCC	*AfupyrG* _R
OHS1992	CACATCACTTTGTTGCTGGCT	*lreA*_5' DF
OHS2010	*GGCTTTGGCCTGTATCATGACTTCA* GAATGAAGCGATGAGTGGGC	*lreA*_Rev with *AfupyrG* tail R
OHS1994	*TTTGGTGACGACAATACCTCCCGAC* ACAAGCGTCATGTAGAGGGA	*lreA*_Rev with *AfupyrG* tail F
OHS1995	GCTCCACCATCGTCCAATTC	*lreA*_3' DR
OHS1996	GCATGTCGACATCGACGC	*lreA*_nested 5' NF
OHS1997	GACACCCTTAGCTGTCCTAGC	*lreA*_nested 3' NR
OHS1998	TGCCAACAAGAGGTTCAAGC	*lreA*_RT_F
OHS1999	CGCTTCTTTCTGCGGGTAAG	*lreA*_RT_R
OHS2740	AATT **GCGGCCGC** CCGTCCTTCTTTAAGGTCGCA	C' *lreA*_*Not*Ι_F
OHS2076	AATT **GCGGCCGC** TGCGTTACGGACCTGCTT	C' *lreA*_*Not*Ι_R
OHS2000	GTTGTCGAAGGAGCGAACC	*lreB*_5' DF
OHS2001	*GGCTTTGGCCTGTATCATGACTTCA* CTGTGCTGGTGATTCTGCG	*lreB*_Rev with *AfupyrG* tail R
OHS2002	*TTTGGTGACGACAATACCTCCCGAC* GCAAGTGCCTCAATGAGGG	*lreB*_Rev with *AfupyrG* tail F
OHS2003	CACTGGGATTGCTTCTCACG	*lreB*_3' DR
OHS2004	CACCACTTACACCTGCCAG	*lreB*_nested 5' NF
OHS2005	CTCTATGCTGCTGCGAACC	*lreB*_nested 3' NR
OHS2006	TGAGGAGAACGAGTCATCCG	*lreB*_RT_F
OHS2007	CCGTAGTGTAGTCCCGTCAA	*lreB*_RT_R
OHS2186	AATT **GCGGCCGC** TAACTTTCGGACTCTCGCCA	C' *lreB*_*Not*Ι_F
OHS2078	AATT **GCGGCCGC** CCGACTGTCATTTGTTCATTATACCAT	C' *lreB*_*Not*Ι_R
OHS0197	TCCCAGCAATCTGCCCTTGC	*AfupyrG*_middle_R
OHS1779	AGGGTCGGAACAGGAGAG	pYES1_F
OHS0405	TATGTCGGTGATGAGGCACA	actin_RT_F
OHS0406	AACACGGAGCTCGTTGTAGA	actin_RT_R

*Italics show the tail sequence, and bold text indicates the restriction sequence.
