# Supplementary material for: Characterization of Blue Light Receptors LreA and LreB in Aspergillus flavus
Source: J Microbiol Biotechnol. 2025 Feb 14;35:e2411054. doi: 10.4014/jmb.2411.11054 (PMC11876014; doi:10.4014/jmb.2411.11054)
Supplement: Supplementary file 1 [file jmb-35-e2411054-supple.pdf]

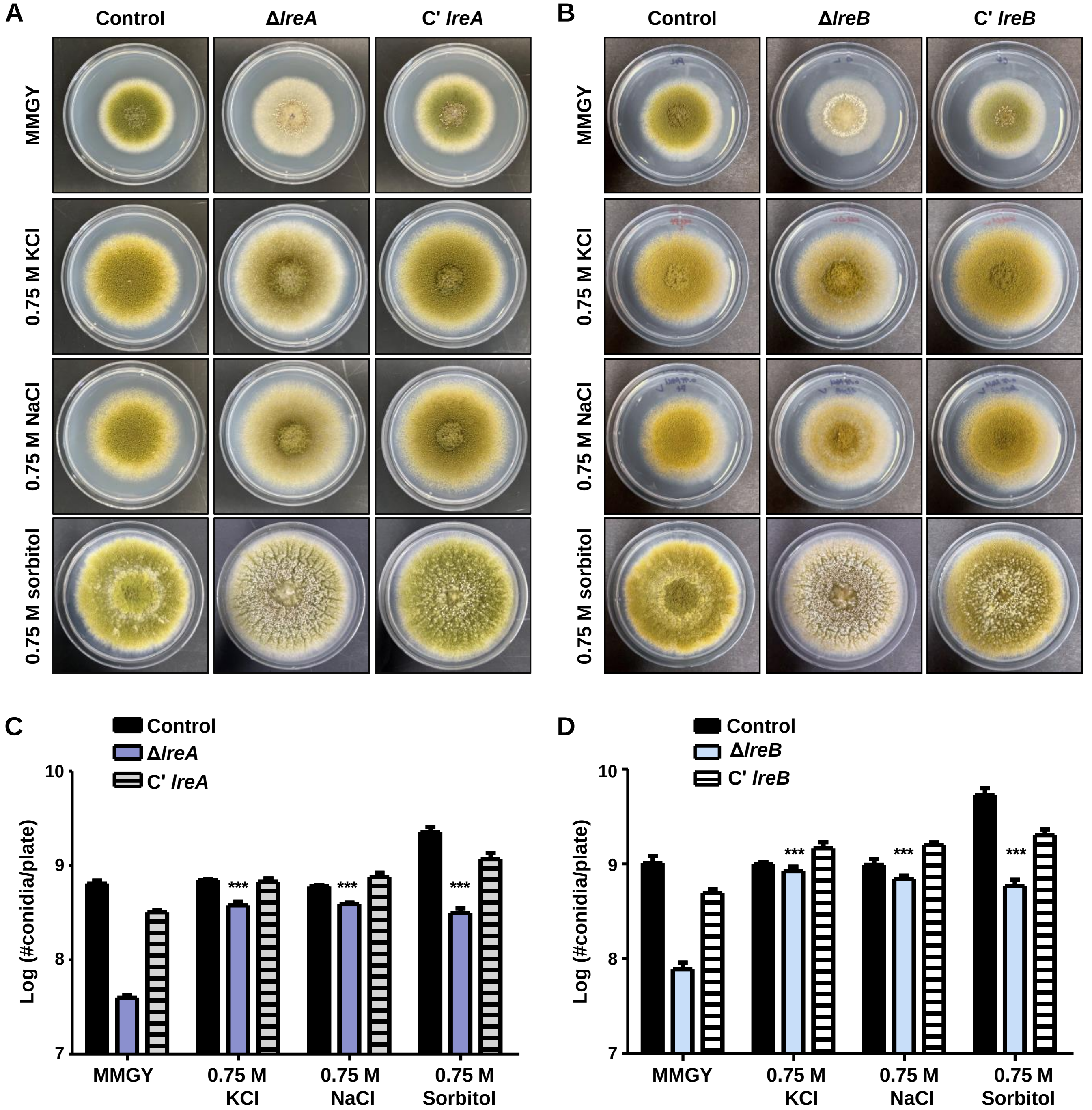

Fig. S1. Stimulation of asexual development through osmotic stimulation in the Δ*lreA* and Δ*lreB* strains. (A) Images of the control, Δ*lreA*, and C' *lreA* strains, point inoculated on solid MMGY media containing 0.75 M KCl, 0.75 M NaCl, or 0.75 M sorbitol, were grown at 37℃ under light conditions for 5 days. (B) Images of the control, Δ*lreB*, and C′ *lreB* strains, which had been point-inoculated on solid MMGY medium with osmotic stimulation, were cultured at 37℃ under light conditions for 5 days. (C) The number of conidia when applying various osmotic stimulations to the control, Δ*lreA*, and the C' *lreA* strains. (D) The number of conidia when stimulating the control, Δ*lreB*, and C′ *lreB* strains to different osmotic induction sources. (MT on MMGY medium vs MT on each osmotic medium, **p* ≤ 0.05; ***p* ≤ 0.01; ****p* ≤ 0.001, *n*=3)

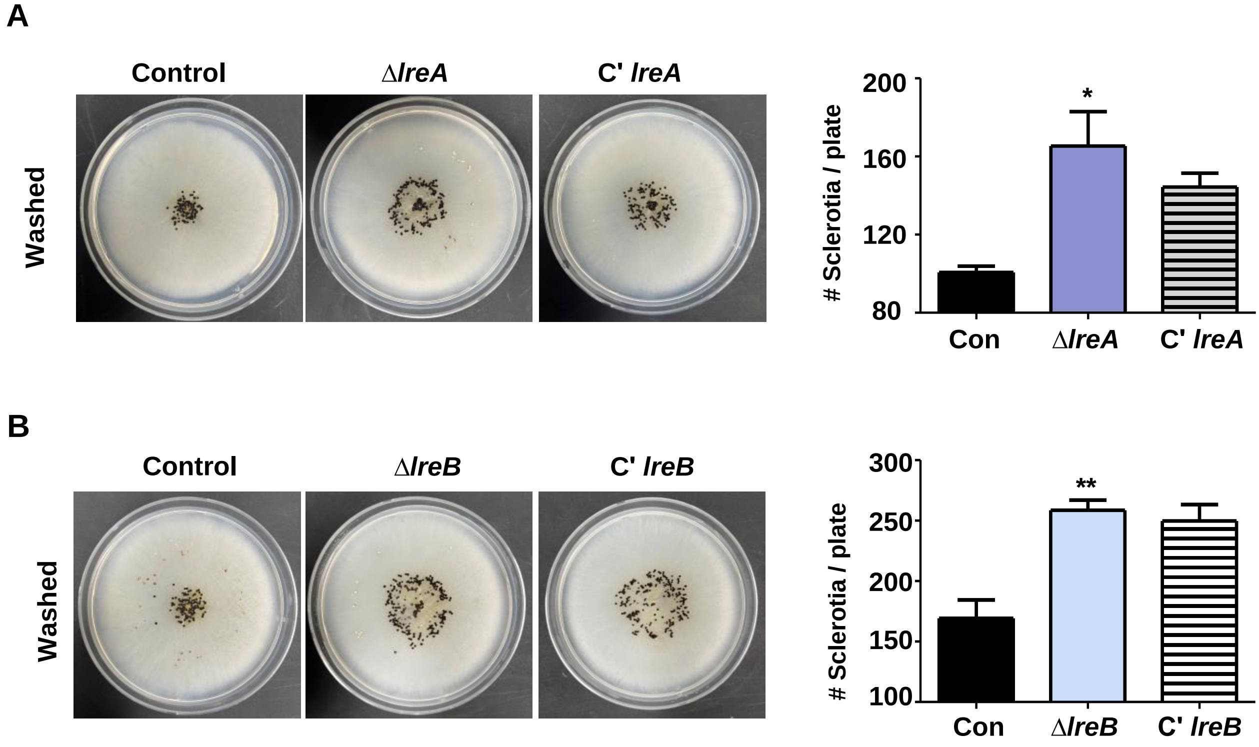

Fig. S2. The effects on fungal growth and sexual development of LreA and LreB *in A. flavus*. (A) Washed plates of the Δ*lreA* strain grown at 37°C under dark conditions for 7 days. The number of sclerotia of the Δ*lreA* strain. (Control vs. Δ*lreA*, *$p \leq 0.05$, n=3). (B) Washed plates of Δ*lreB* grown at 37°C under dark conditions for 7 days. The number of sclerotia of the Δ*lreB* strains (control vs. Δ*lreB*, **$p \leq 0.01$, n=3).

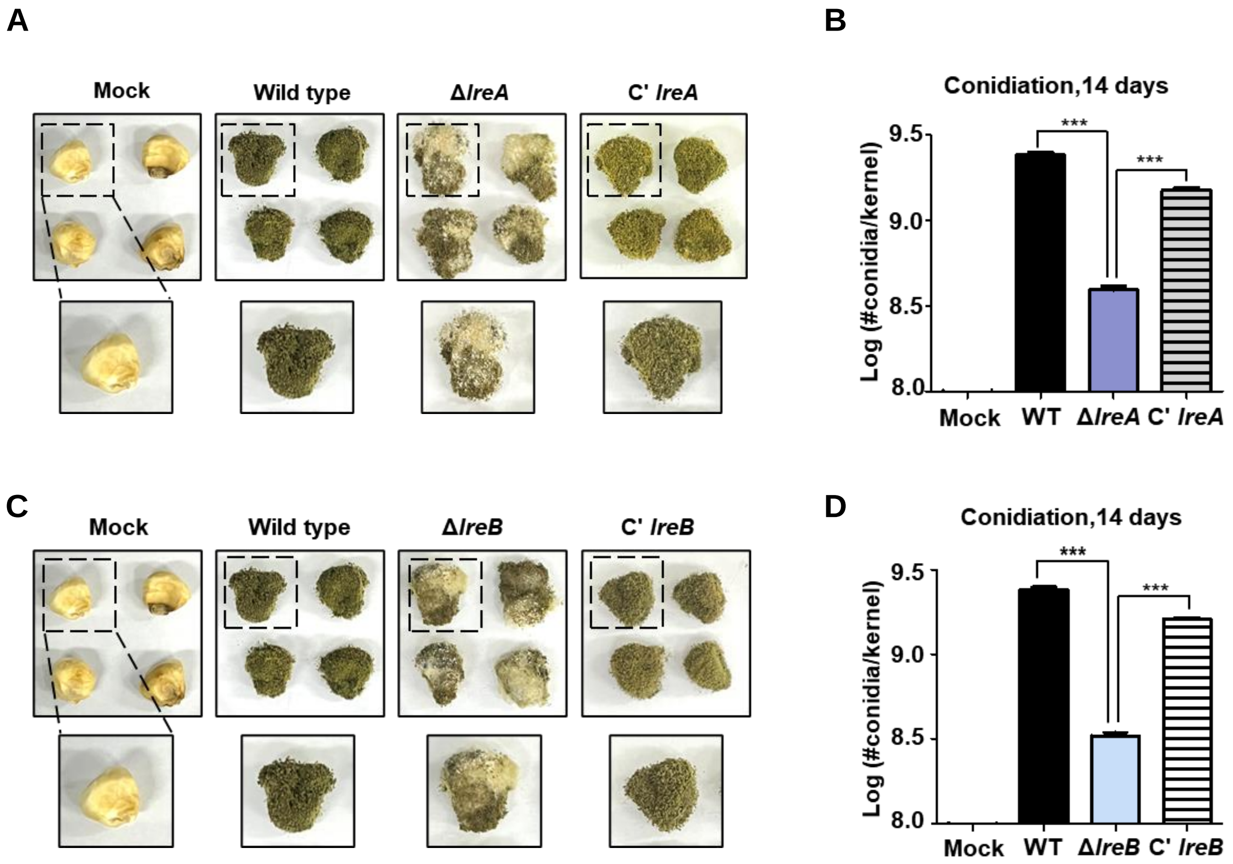

Fig. S3. Role of *lreA* and *lreB* in kernel infection. (A) Results obtained after growth at 30°C under light conditions for 14 days following kernel infection with the wild-type, Δ*lreA*, and C' *lreA* conidia. The mock refers to the kernel itself, which was not inoculated with any conidial strain. The others were inoculated with the wild-type, Δ*lreA*, and C' *lreA* strains. (B) Counting and comparison of the number of conidia growing on each maize kernel for 14 days (WT vs. Δ*lreA*, ***$p \leq 0.001$, n=3). (C) These results were obtained after the kernels were infected with the wild-type, Δ*lreB*, and C' *lreB* conidia and grown at 30°C under light conditions for 14 days. The mock represents uninfected kernels, while the others were grown with the inoculation of the wild-type, Δ*lreB*, and C' *lreB* conidia for 14 days. (D) The conidia of each strain were cultured on kernels for 14 days, and fungal colonization was compared (WT vs. Δ*lreB*, ***$p \leq 0.001$, n=3).

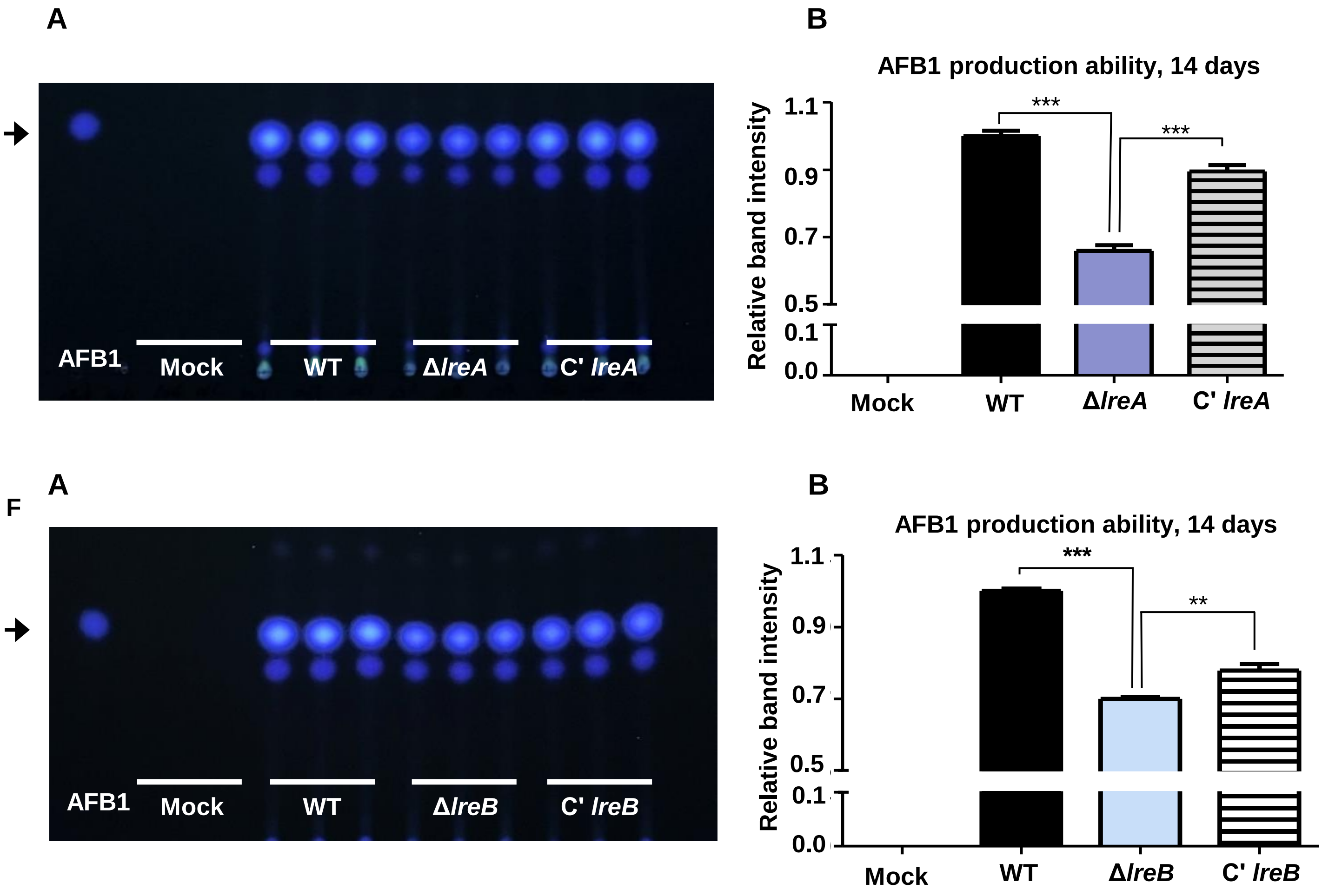

Fig. S4. Role of *lreA* and *lreB* in aflatoxin B1 production. Image showing the production of aflatoxin B1 extracted from the kernel samples infected with control, Δ*lreA*, and C' *lreA* conidia for 14 days of cultivation. (B) Relative band intensity of aflatoxin B1 according to the TLC result in (A). Error bars indicate the standard error of the mean from three biological replicates (WT vs. Δ*lreA*, ***$p \leq 0.001$, n=3). (C) Image showing the production of aflatoxin B1 extracted from the kernel samples infected with control, *ΔlreB,* and C' *lreB* conidia for 14 days of cultivation. (D) Relative band intensity of aflatoxin B1 according to the TLC result in (C). Error bars indicate the standard error of the mean from three biological replicates (WT vs. *ΔlreB, ,* **$p \leq 0.01$; ***$p \leq 0.001$, n=3).
